# Mir24-2-5p suppresses the osteogenic differentiation with Gnai3 inhibition presenting a direct target via inactivating JNK-p38 MAPK signaling axis

**DOI:** 10.7150/ijbs.60536

**Published:** 2021-10-17

**Authors:** Li Meng, Lichan Yuan, Jieli Ni, Mengru Fang, Shuyu Guo, Huayang Cai, Jinwei Qin, Qi Cai, Mengnan Zhang, Fang Hu, Junqing Ma, Yang Zhang

**Affiliations:** 1 Jiangsu Key Laboratory of Oral Diseases, Nanjing Medical University, Nanjing 210029, China.; 2 Department of Orthodontics, Affiliated Hospital of Stomatology, Nanjing Medical University, Nanjing 210029, China.; 3 Department of Stomatology, Changhai Hospital, Navy Medical University, Shanghai 200433, China.

**Keywords:** Craniofacial deformity, Osteogenic differentiation, MicroRNA, G protein family, Gene expression regulation

## Abstract

**Background:** Congenital anomalies are increasingly becoming a global pediatric health concern, which requires immediate attention to its early diagnosis, preventive strategies, and efficient treatments. Guanine nucleotide binding protein, alpha inhibiting activity polypeptide 3 (Gnai3) gene mutation has been demonstrated to cause congenital small jaw deformity, but the functions of Gnai3 in the disease-specific microRNA (miRNA) upregulations and their downstream signaling pathways during osteogenesis have not yet been reported. Our previous studies found that the expression of Mir24-2-5p was significantly downregulated in the serum of young people with overgrowing mandibular, and bioinformatics analysis suggested possible binding sites of Mir24-2-5p in the Gnai3 3'UTR region. Therefore, this study was designed to investigate the mechanism of Mir24-2-5p-mediated regulation of Gnai3 gene expression and explore the possibility of potential treatment strategies for bone defects.

**Methods:** Synthetic miRNA mimics and inhibitors were transduced into osteoblast precursor cells to regulate Mir24-2-5p expression. Dual-luciferase reporter assay was utilized to identify the direct binding of Gnai3 and its regulator Mir24-2-5p. Gnai3 levels in osteoblast precursor cells were downregulated by shRNA (shGnai3). Agomir, Morpholino Oligo (MO), and mRNA were microinjected into zebrafish embryos to control *mir24-2-5p* and *gnai3* expression. Relevant expression levels were determined by the qRT-PCR and Western blotting. CCK-8 assay, flow cytometry, and transwell migration assays were performed to assess cell proliferation, apoptosis, and migration. ALP, ARS and Von Kossa staining were performed to observe osteogenic differentiation. Alcian blue staining and calcein immersions were performed to evaluate the embryonic development and calcification of zebrafish.

**Results:** The expression of Mir24-2-5p was reduced throughout the mineralization process of osteoblast precursor cells. miRNA inhibitors and mimics were transfected into osteoblast precursor cells. Cell proliferation, migration, osteogenic differentiation, and mineralization processes were measured, which showed a reverse correlation with the expression of Mir24-2-5p. Dual-luciferase reporter gene detection assay confirmed the direct interaction between Mir24-2-5p and Gnai3 mRNA. Moreover, in osteoblast precursor cells treated with Mir24-2-5p inhibitor, the expression of Gnai3 gene was increased, suggesting that Mir24-2-5p negatively targeted Gnai3. Silencing of Gnai3 inhibited osteoblast precursor cells proliferation, migration, osteogenic differentiation, and mineralization. Promoting effects of osteoblast precursor cells proliferation, migration, osteogenic differentiation, and mineralization by low expression of Mir24-2-5p was partially rescued upon silencing of Gnai3.* In vivo*, *mir24-2-5p* Agomir microinjection into zebrafish embryo resulted in shorter body length, smaller and retruded mandible, decreased cartilage development, and vertebral calcification, which was partially rescued by microinjecting *gnai3* mRNA. Notably, quite similar phenotypic outcomes were observed in *gnai3* MO embryos, which were also partially rescued by *mir24-2-5p* MO. Besides, the expression of phospho-JNK (p-JNK) and p-p38 were increased upon Mir24-2-5p inhibitor treatment and decreased upon shGnai3-mediated Gnai3 downregulation in osteoblast precursor cells. Osteogenic differentiation and mineralization abilities of shGnai3-treated osteoblast precursor cells were promoted by p-JNK and p-p38 pathway activators, suggesting that Gnai3 might regulate the differentiation and mineralization processes in osteoblast precursor cells through the MAPK signaling pathway.

**Conclusions:** In this study, we investigated the regulatory mechanism of Mir24-2-5p on Gnai3 expression regulation in osteoblast precursor cells and provided a new idea of improving the prevention and treatment strategies for congenital mandibular defects and mandibular protrusion.

## Introduction

Osteogenic differentiation has been identified to play critical roles in promoting bone repair and reconstructions in patients with bone development anomalies 1. Auriculo-condylar syndrome (ACS) is a craniofacial malformation caused by disruptions of embryonic development of the first and second pharyngeal arches, particularly involving the development of the mandible and ear 2. The dominant characteristic features of ACS include micrognathia, small mandibular condyle, and question-mark ear. Severe small mandible ACS patients usually need bilateral mandibular ramus enlargement, which could be obtained from distraction osteogenesis or orthognathic surgery, which is time-consuming and expensive 3. On the contrary, excessive bone formation lays behind mandibular prognathism which is a maxillofacial disorder characterized by an abnormal forward projection of the lower jaw 4. Given that both ACS and mandibular prognathism involve defects in osteogenic differentiation, we sought to investigate the regulatory mechanism of osteogenic differentiation to facilitate the development of potential treatment strategies for congenital bone defects and abnormal bone overgrowth.

Genomic alterations in Guanine nucleotide-binding protein, alpha inhibiting activity polypeptide 3 (GNAI3) on chromosome 1p13, phospholipase C, beta 4 (PLCB4) on chromosome 20p12.3-p12.2, and endothelin 1 (EDN1) on chromosome 6p24 have reportedly been identified as responsible for congenital craniofacial deformations in > 90 % of examined ACS cases. G-proteins are ubiquitously expressed in cells and are typically composed of α, β, and γ subunits. Genes encoding a variety of Gα proteins can be grouped into Gαs, Gαi, Gαq, and Gα12 subfamilies according to their structural and functional similarities 5. Studies have shown that GNAI3, which belongs to Gαi subfamily, participates in several critical biological processes and regulates many cellular activities, including proliferation, differentiation, apoptosis, and migration 5, 6. Importantly, GNAI3 has been found to hold a potential correlation with cartilage degeneration in the rat model of osteoarthritis 7. However, the mechanistic connection of GNAI3 with osteogenesis remains poorly understood 8. The homeobox genes distal less homeobox 5 (DLX5) and distal less homeobox 6 (DLX6) play critical functions in cranial neural crest cells (NCCs) and drive the primary normal lower jaw patterning. Notably, osteoblast precursor cells isolated from ACS probands have exhibited significant expression reductions of DLX5 and DLX6 9. Moreover, mice embryos with a combined loss of both Dlx5 and Dlx6 have displayed an ACS-like phenotype in the lower jaw, which is maxillary-like-structure 10. Besides, PLCB4 and GNAI3 play key roles as the signal modulators in the EDN1-DLX5/DLX6 network axis to pattern the lower jaw and other pharyngeal skeletal structures 9, 11. These suggest that GNAI3 may play an indispensable role in the early embryonic bone osteogenesis.

MicroRNAs (miRNAs) have been shown to maintain homeostasis between bone formation and resorption during osteogenesis by positively or negatively modulating expressions of associated genes at the post-transcriptional level 12. Notably, Mir24 has been identified as a potential biomarker for bone metabolism as both circulating and tissue-specific microRNA. Increased expression of plasma Mir24-3p has been detected in cases with osteoporotic fractures induced by the systemic reduction of bone strength 13. It has been found that five freely circulating miRNAs, including Mir24-3p, are significantly upregulated in both serum and bone tissue in patients with osteoporotic fractures 14. Besides, we have previously reported the downregulation of 49 serum miRNAs, including Mir24-2-5p, in young patients with mandibular prognathism with at least two-fold expression change by miRNA microarray assay 15.

In this study, we found reverse expression patterns for Mir24-2-5p and Gnai3 during the osteogenic differentiation of osteoblast precursor cells. We hypothesized that Mir24-2-5p might regulate the osteogenesis of osteoblast precursor cells by targeting Gnai3. Furthermore, unveiling the key molecular mechanism involving the Mir24-2-5p-Gnai3 axis may lead to the paradigm shift in our understanding ACS and mandibular retraction, as well as providing new ideas for the prevention and treatment of ACS and mandibular hypoplasia pathogenesis thereby facilitating bone tissue engineering and identification of novel therapeutic interventions in treating bone defects.

## Methods and Materials

### Animals

30 5-day-old mice (C57BL/6J) were obtained from the Animal Center of Nanjing Medical University (Nanjing, China). 300 wild-type zebrafish (*Danio rerio*) embryos were maintained and raised under standard conditions 16. The wild-type Lon AB line was provided by the Model Animal Research Center of Nanjing University (Nanjing, China). All animal experiments were performed according to the protocols approved by the Experimental Animal Care Committee of Nanjing Medical University.

### Primary osteoblast precursor cells

The calvarial bones were isolated from postnatal 5-day mice and cut into 0.1 cm×0.1 cm×0.1 cm pieces. Calvaria pieces were cultured in α-MEM (Gibco, #12561072) supplemented with 10% fetal bovine serum (FBS, Gibco, #12483020) and 1% penicillin-streptomycin (PS) (Gibco, #10378016) for the emigration of primary osteoblast precursor cells, which were then incubated at 37 °C with 5 % CO_2_. The medium was changed every three days until cells reached 90 % confluency 17.

### Mir24-2-5p-specific mimics and inhibitors

Mir24-2-5p mimics (mimics, chemically modified double-stranded RNA, sense strand: 5′ - GUGCCUACUGAGCUGAAACAGU - 3′, antisense strand: 5' - ACUGUUUCAGCUCAGUAGGCAC - 3', RIBO technology) and inhibitor (inhibitor, single-stranded RNA with the sequence 5′ - ACUGUUUCAGCUCAGUAGGCAC - 3′, RIBO technology) were transfected into primary osteoblast precursor cells using a riboFECT CP Transfection Kit (RIBO technology) following the manufacturer's protocol. In brief, primary osteoblast precursor cells were cultured in six-well plates to 70% confluency and then transfected with mimics, mimics negative control (NC, 5' - UCACAACCUCCUAGAAAGAGUAGA - 3', RIBO technology), inhibitor and inhibitor negative control (NC, 5' - UCACAACCUCCUAGAAAGAGUAGA - 3', RIBO technology). Followed by 24 h of culture, cells were subjected to an additional 48 h of culture in a complete αMEM medium supplemented with 10% FBS and 1% PS.

### qRT-PCR assay

Total RNA was extracted using an RNA isolation kit (BioTeke, Beijing, China). Reverse transcription was conducted using PrimeScript RT reagent kit (Vazyme, Nanjing, China). Relative expression levels of Mir24-2-5p and target genes (Gnai3, runt-related transcription factor 2 Runx2, Osterix Osx, Osteopontin Opn, and Osteocalcin Ocn) were examined by qRT-PCR assay using ChamQ SYBR qPCR Master Mix (Vazyme, Nanjing, China) on the ABI-7300 Real-Time PCR System (Applied Biosystems, CA, USA). The thermal cycling profile was listed as follows:

The sequences of primer pairs used are listed in **Supplementary Table S1**. The quantitative analysis was performed by 2^-∆∆CT^ method relative to the U6 small nucleus RNA for Mir24-2-5p and GAPDH.

### CCK-8 assay

The proliferation of primary osteoblast precursor cells was estimated by Cell Counting Kit-8 (CCK-8; Dojindo Kagaku Co., Kumamoto, Japan) according to the manufacturer's protocol. Briefly, the primary osteoblast precursor cells were seeded at ~1×10^4^ cells/well in a 96-well plate. Followed by 24 h of incubation (day 0), 10 µl of CCK-8 reagent was added to each well. After 2 h of incubation, optical density (OD) was measured using a microplate reader at 450 nm.

### Flow cytometry assay

To examine cell cycle arrest, the primary osteoblast precursor cells were collected and fixed with 70% ethanol. The cells were then stained with 20 mg/ml propidium iodide (PI) for 1 h. Flow cytometry (FACSCalibur, BD Biosciences) was then used to determine the cell cycle distribution according to the manufacturer's instructions. FlowJo V7 software (Tree Star, Oregon, USA) was used for analysis. To detect cellular apoptosis rate, the cells were digested with trypsin, resuspended in PBS, and stained with the Annexin V-FITC/PI kit (Nanjing KeyGen Biotech co., Ltd., Nanjing, China) according to the manufacturer's instructions. Quantitative analysis was performed by flow cytometry (BD FACSverse™) and analyzed with FlowJo V7.

### Alkaline phosphatase (ALP) activity assay

BCIP/NBT Alkaline Phosphatase color development kit (Beyotime Institute of Biotechnology) was used for ALP analysis according to the manufacturer's protocol. In brief, the primary osteoblast precursor cells were seeded in 12-well plates at 4×10^4^ cells/well and cultured in osteogenic medium (α-MEM +10 % FBS +1% PS +100 µM ascorbic acid +2 mM 2-glycerophosphate + 10 nm dexamethasone) for 5 days. The stained culture plates were imaged by microscope (DMIL LED) and quantified by Image J v.1.45 software (National Institutes of Health, USA).

### Alizarin red-Staining (ARS) mineralization assay

2% ARS reagent (Beyotime Institute of Biotechnology, Shanghai, China) was used to detect matrix mineralization. The primary osteoblast precursor cells were seeded in 12-well plates at 4×10^4^ cells/well and cultured in osteogenic medium (α-MEM +10 % FBS +1 % PS +100 µM ascorbic acid +2 mM 2-glycerophosphate + 10 nm dexamethasone) for 14 days and then stained with 2% ARS solution. The mineralized nodules were imaged by microscope (DMIL LED) and quantified by Image J software (National Institutes of Health, USA).

### Von Kossa staining

Von Kossa staining was performed to visualize mineralized extracellular matrix with Von Kossa staining Kit (Genmed, Shanghai, China). In brief, the cells were fixed with 4% PFA for 15 min after 14 days of incubation with osteogenic medium, rinsed with distilled water, and then stained with CENMED staining solution (containing 1% silver nitrate solution), followed by exposure to ultraviolet light for 40 minutes. The black deposits of calcium were imaged by microscope (DMIL LED).

### Western blotting analysis

Total protein extracted from primary osteoblast precursor cells using RIPA buffer with protease inhibitor phenylmethanesulfonyl fluoride (PMSF) was quantified with a BCA Protein Assay Kit (Beyotime, Jiangsu, China). A total of 20 µg of protein was denatured and separated using 10% SDS-PAGE and then transferred to polyvinylidene fluoride (PVDF) membranes, blocked in 5% nonfat milk in tris-buffered saline/Tween-20 for 2 h, and incubated with primary antibodies overnight at 4 °C. Primary antibodies against GNAI3 (ab154024, Abcam), RUNX2 (ab76956, Abcam), OSX (ab22552, Abcam), OPN (ab63856, Abcam), OCN (ab93876, Abcam), ERK (#4694, Cell Signaling Technology), p-ERK (#4370, Cell Signaling Technology), JNK (#9252, Cell Signaling Technology), p-JNK (#4668, Cell Signaling Technology), p38 (#9212, Cell Signaling Technology), p-p38 (#9211, Cell Signaling Technology, Boston, USA) and GAPDH (AP0063, Bioworld) were used. Subsequently, the membranes were incubated with HRP-conjugated secondary antibodies (1:8000, ZB-2301, Origene Technologies) for 1 h at room temperature. Protein bands were visualized with electrochemiluminescence substrate solution (GE Healthcare Life Sciences) and imaged by Micro chemiluminescence system 4.2 (DNR Bio-Imaging Systems, Ltd., Jerusalem). Semi-quantitative analysis was conducted with Image J v.1.45 software.

### Transwell migration assay

The primary osteoblast precursor cells were placed into the upper chambers (Corning, USA), αMEM medium containing 10% FBS was added to the lower chambers. Cells were then incubated at 37 °C with 5% CO_2_ for 24 h or 48 h, fixed with 4% PFA, and stained with 0.5% crystal violet (Sigma) for 5 min. Then, the migrated cells were imaged using the microscope and quantified with Image J v.1.45 software.

### Dual-luciferase reporter assay

The reporter vector pLUC-Gnai3 3'UTR wt was formed by cloning Gnai3 cDNA, which contains the binding site of Mir24-2-5p, into the pLUC Dual-luciferase miRNA Target Expression Vector (Promega). The vector pLUC-Gnai3 3'UTR mutant was inserted by the mutant Gnai3, which contains point mutations of the binding site. The primers were used as followed: Gnai3-F XhoI: ATCGCTCGAGCAATTCCCACAATGTAGGGGC, Gnai3-R BamHI: ATCGGGATCCGCCTTCAATCCACCGAGGAA for Gnai3 3'UTR wt reporter plasmid, Gnai3 -MF: TTTCTATCCGTAAATCAAAATGTGGGGGGATTAAAC, Gnai3-MR: TGATTTACGGATAGAAATGTTCTTCAATAAACATTAGTGA for Gnai3 3'UTR mutant reporter plasmid. HEK 293T cells were seeded into 96-well plates at a density of 2 × 10^4^ cells/well and incubated for 24 h. The cells were co-transfected with Mir24-2-5p mimics or Mir24-2-5p mimics control together with 200 ng Gnai3 3'UTR wt or Gnai3 3'UTR mutant reporter plasmid using Lipofectamine-2000 transfection reagent according to the manufacturer's instruction. After 48 h of incubation, Luciferase Reporter Assay System (Promega, Cat #E1980) was used to detect luciferase activities.

### Lentivirus transfection

Recombinant lentiviral LV3 vectors of shRNA targeting mouse Gnai3 mRNA (shGnai3, 5'-GGTTTACAGACACTTCAAT-3') and negative control lentivirus shGFP (shCtrl, 5'- TTCTCCGAACGTGTCACGT-3') were purchased from GenePharma (Shanghai, China). When the primary osteoblast precursor cells grew to 70% confluence, lentiviral particles (MOI = 50) were added to the medium in the presence of 1 μg/ml polybrene. The medium was refreshed with fresh complete medium after 24 h. After 72 h of transduction, transduced cells exhibited GFP expression, which was detected by fluorescence microscopy (DMI3000B; Leica Microsystems GmbH).

### Micro-injections

Morpholino oligos (MOs) were acquired from Gene Tools (Philomath, USA). The MOs used in this study are as follows: MO targeting at *mir24-2-5p*: *mir24-2-5p* MO, 5'-CCTGTTCCTGCTGAACTGAGCCAG-3', MO targeting at *gnai3*: *gnai3* MO, 5'-TCAGTGCTTAACGTGCAACCCATTT-3' and standard control MO: *control* MO, 5'-CTAAAAGCA-GCAGGAGGCGATTCAT-3', All MOs were microinjected into embryos at the 1-2 cell stage at a dose of 10 pg/embryo.

Mir24-2-5p Agomir (5'-GUGCCUACUGAGCUGAAACAGU-3' and 5'-UGUUUCAGCUCAGUAGGCACUU-3') and Negative Control (5'-UUUGUACUACACAAAAGUACUG-3' and 5'-GUACUUUUGUGUAGUACAAATT-3'); were designed and produced by Ribobio (Guangzhou, China). They were injected into zebrafish embryos at 1-2 cell stage with a micro-injector (Applied Scientific Instrumentation MPPI-3, Eugene, USA) at 200 pg/embryo.

The sequence of *gnai3* was obtained from NCBI Gene Database. *gnai3* cDNA was cloned and ligated into the pXT7 plasmid, which was linearized by XbaI and transcribed with the mMESSAGE mMACHINE T7 kit (Ambion, #AM1344) for *gnai3* mRNA. *gnai3* mRNA was injected into 1-2 cell stage zebrafish embryos at a dose of 50 pg/embryo.

### Alcian blue staining

120-hour post-fertilization (hpf) zebrafish embryos were fixed with 4 % PFA for 48 h and stained with alcian blue solution (Sigma, #A5268) as previously described 18. The embryos were digested with a KOH/glycerol series until they were transparent and finally conserved in 100% glycerol. The length of line A/B/C was measured as described previously 16, 19. Line A is regarded as a baseline for later measurements. Line B is the distance from line A to the front of the Meckel's cartilage. Line C is the distance from line A to the front of the ceratohyal. The ratios of line B divided by line A (B/A) and line C divided by line A (C/A) were quantified.

### Calcein Immersions

9 dpf zebrafish were placed in the cultured medium for 1 h, immersed in 0.2 % calcein (pH = 7) solution (Sigma) in dark for 3-10 min. The medium was rinsed several times with fresh water to remove the dye and then stood again for 1 h. The embryos were anesthetized with 0.04 g/L tricaine and imaged with a fluorescence microscope. The fluorescence intensity and positive area of the first 3 vertebrae were measured using the PlotProfile tool in Image J software.

### Statistical analysis

All the experiments were carried out at least three times respectively. All data were expressed as the mean standard error of the mean (S.E.M.). Statistical analyses were assessed using Student's *t*-test by GraphPad PRISM software (ver.8.3.0, La Jolla, CA). The result with p < 0.05 was considered statistically significant.

## Results

### Mir24-2-5p negatively regulates osteoblast precursor cells differentiation

**Figure 1:**
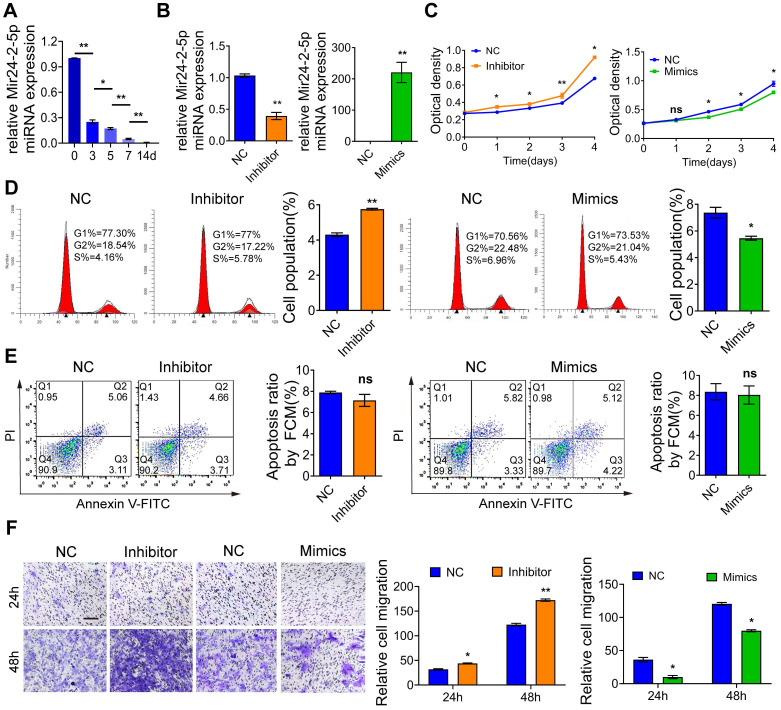
** Mir24-2-5p negatively regulates osteoblast precursor cells cellular proliferation and migration. (A)** Expression of Mir24-2-5p determined by qRT-PCR at day 0, 3, 5, 7, and 14 of osteogenic differentiation of osteoblast precursor cells. n=5. **(B)** Expression of Mir24-2-5p determined by qRT-PCR after transfection of Mir24-2-5p Inhibitor and Mir24-2-5p Mimics. n=4. **(C)** CCK-8 assay over the course of 4 days. n=4. **(D)** Cell cycle analysis performed by flow cytometry in Inhibitor and Mimics groups. n=4. **(E)** Apoptosis analysis performed by flow cytometry. n=4. **(F)** Transwell migration assay testing of osteoblast precursor cells at 24 and 48 hours in Inhibitor and Mimics groups. Bar = 100 μm. n=4. Data expressed as mean ± S.E.M. *p < 0.05, **p < 0.01, ns, not significant.

**Figure 2:**
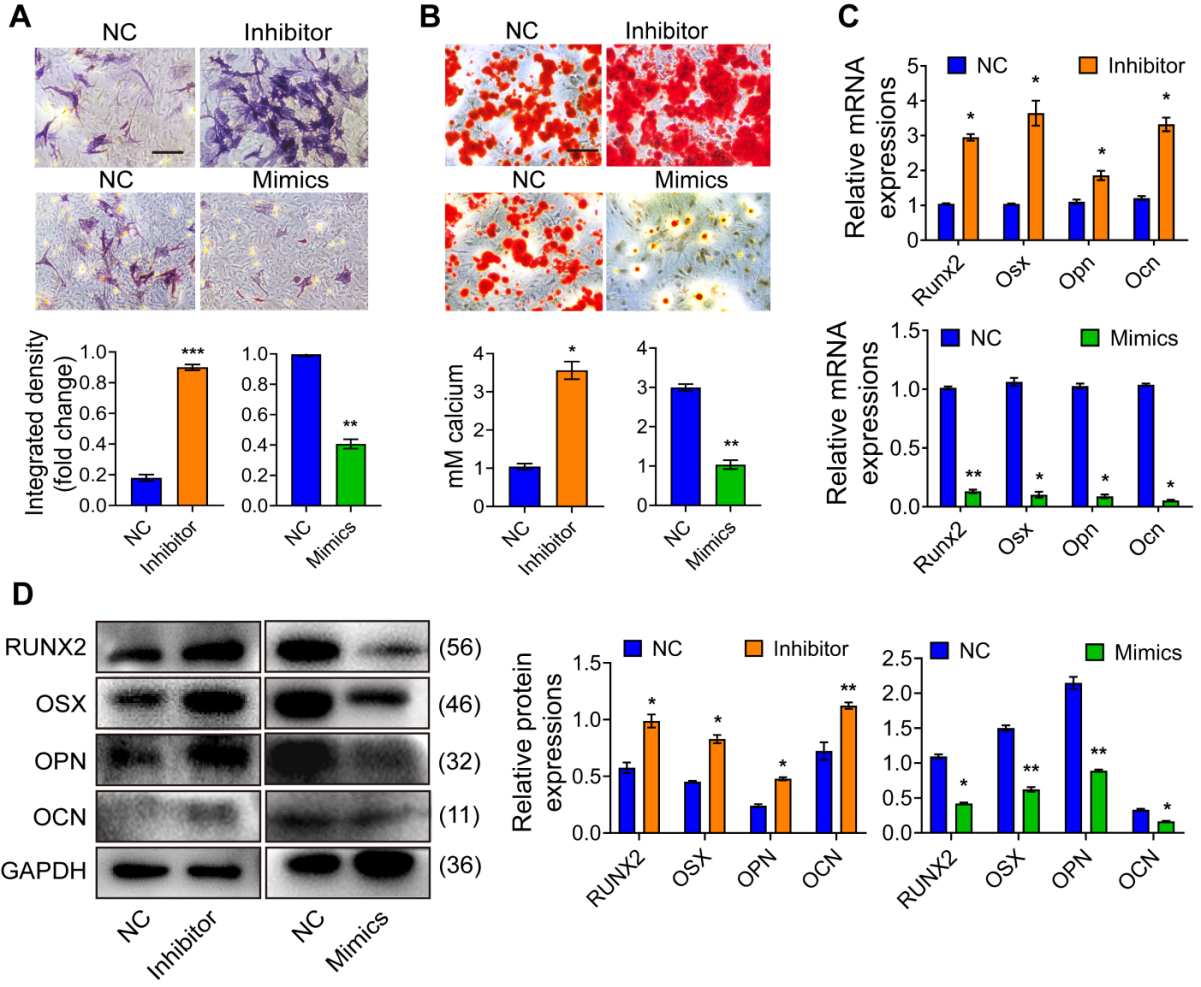
** Mir24-2-5p negatively regulates osteogenic differentiation of osteoblast precursor cells. (A)** ALP staining observed after 5 days' mineralization. Bar = 100 μm. n = 4. **(B)** ARS staining observed after 14 days' mineralization. Bar = 100 μm. n = 4. **(C)** Expression levels of osteogenic related genes (Runx2, Osx, Opn, Ocn) assessed by qRT-PCR. n = 4. **(D)** Expressions of osteogenic markers (RUNX2, OSX, OPN, OCN) assessed by Western blotting. n = 4. Data expressed as mean ± S.E.M. *p < 0.05, **p < 0.01.

Total RNA was extracted separately from osteoblast precursor cells after mineralization induction for 0d, 3d, 5d, 7d, and 14d for the qRT-PCR-based expression analysis. We found that the expression of Mir24-2-5p was gradually decreased over time** (Fig. 1A)**, indicating that Mir24-2-5p might negatively regulate the process of osteogenic differentiation. Next, the osteoblast precursor cells were transfected with the negative control (NC), or Mir24-2-5p inhibitor (Inhibitor) or Mir24-2-5p mimics (Mimics), followed by qRT-PCR analysis to confirm Mir24-2-5p expression level in osteoblast precursor cells. The results showed that Mir24-2-5p was about 60 % downregulated (~two-fold) by treatment with the inhibitor, while the Mimics treatment induced its expression by >200-fold **(Fig. 1B)**. The effect of Mir24-2-5p on cell proliferation was determined by CCK-8 and flow cytometry analyses revealing that the proliferation of osteoblast precursor cells with a low level of Mir24-2-5p was increased; However, the proliferation of osteoblast precursor cells with Mir24-2-5p overexpression was decreased **(Fig. 1C, D)**. Furthermore, flow cytometry analysis showed that the population of apoptotic cells remained unchanged irrespective of the Mir24-2-5p expression levels, suggesting that Mir24-2-5p expression modulation was not involved in the osteoblast precursor cells apoptosis **(Fig. 1E)**. Next, to detect the effects of Mir24-2-5p expression on cell migration, a transwell migration assay was performed, which showed that the migration ability of the Inhibitor group was increased compared to NC group, while the migration ability of the Mimics group was significantly decreased compared to NC group **(Fig. 1F)**. To explore the regulation of Mir24-2-5p in osteogenic differentiation, ALP staining and ARS staining were performed after 5 days and 14 days of mineralization induction, which showed that the ALP activity and calcium nodule formation of the Inhibitor group were significantly increased compared with those of the NC group, while those of the Mimics group was significantly decreased compared with NC group **(Fig. 2A-B)**. In addition, Von Kossa staining was carried out to confirm that osteoblast precursor cells were fully differentiated and producing mineralized extracellular matrix, which showed consistent results with ALP and ARS staining **(Fig. S1A-B)**, suggesting that Mir24-2-5p has a negative regulatory effect on osteogenic differentiation of osteoblast precursor cells. To further validate this phenomenon, we analyzed the mRNA and protein levels of four osteoblast precursor cells differentiation markers, namely RUNX2, OSX, OPN, and OCN by qRT-PCR and WB analyses respectively. The qRT-PCR analysis showed a significant increase in mRNA expression of osteoblast precursor cells differentiation marker genes in the Inhibitor group, and a significant decrease in the Mimics group **(Fig. 2C)**. Consistently, immunoblotting results exhibited increased protein expressions of differentiation-related genes in the Inhibitor group and subsequent decrease in the Mimics group **(Fig. 2D)**. Together, these results suggested that Mir24-2-5p inhibition accelerated osteogenic differentiation of osteoblast precursor cells, while its stimulation by miRNA mimics suppressed osteogenic differentiation process.

### Mir24-2-5p targets 3'UTR of Gnai3

**Figure 3:**
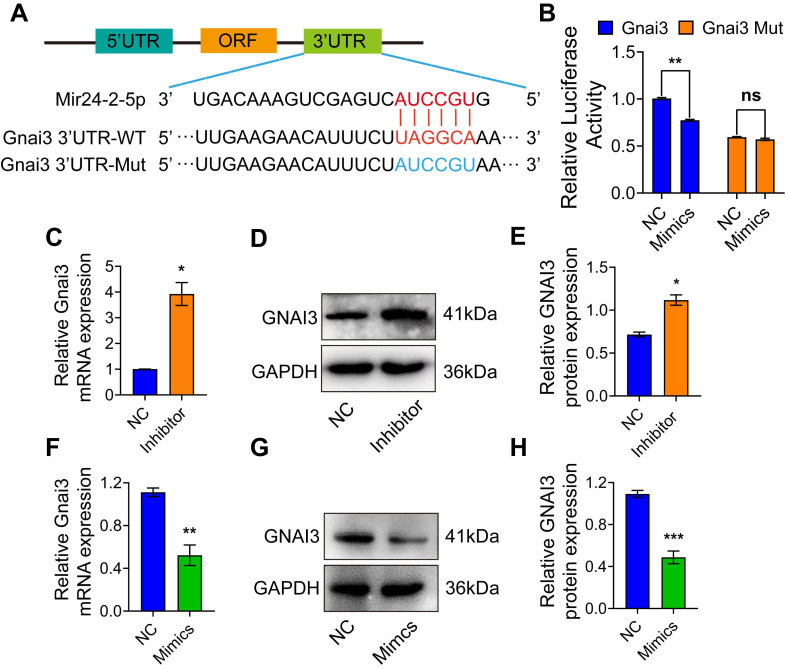
** Mir24-2-5p targets 3'UTR of Gnai3. (A)** The structure diagram of the Dual-luciferase reporter vector assay. **(B)** Quantitative analysis of Dual-luciferase reporter assay. n = 4. **(C)** Expression of Gnai3 assessed by qRT-PCR after transfection of Mir24-2-5p Inhibitor. n = 4. **(D)** Expression of GNAI3 assessed by Western blotting after transfection of Mir24-2-5p Inhibitor. **(E)** Quantitative analysis of Western blotting of (D). n = 4. **(F)** Expression of Gnai3 assessed by qRT-PCR after transfection of Mir24-2-5p Mimics. n = 4. **(G)** Expression of GNAI3 assessed by Western blotting after transfection of Mir24-2-5p Mimics.** (H)** Quantitative analysis of Western blotting of (G). n = 4. Data expressed as mean ± S.E.M. *p < 0.05, **p < 0.01, ***p < 0.001, ns, not significant.

To identify the Mir24-2-5p target genes, we employed the target gene prediction tool Targetscan (http://www.targetscan.org), which identified a potential binding sequence for Mir24-2-5p at the 3'UTR of Gnai3 **(Fig. 3A)**. Dual-luciferase reporter gene assay showed that the luciferase activity in the cells co-transfected with Mir24-2-5p mimics and wt Gnai3 plasmid was decreased compared with that of control, while there was no significant difference in the cells co-transfected with Mir24-2-5p mimics and 3'UTR mutant Gnai3 plasmid, which confirmed the mechanistic cross-talk between Mir24-2-5p and Gnai3 **(Fig. 3B)**. Moreover, qRT-PCR and WB analyses showed significantly increased expressions of Gnai3 at both mRNA and protein levels in osteoblast precursor cells with a low level of Mir24-2-5p expression **(Fig. 3C-E)** and significantly decreased expressions of Gnai3 in osteoblast precursor cells with a high level of Mir24-2-5p expression** (Fig. 3F-H)**, suggesting that Mir24-2-5p negatively regulates the expression of Gnai3 in osteoblast precursor cells.

### Gnai3 stimulates osteogenic differentiation

**Figure 4:**
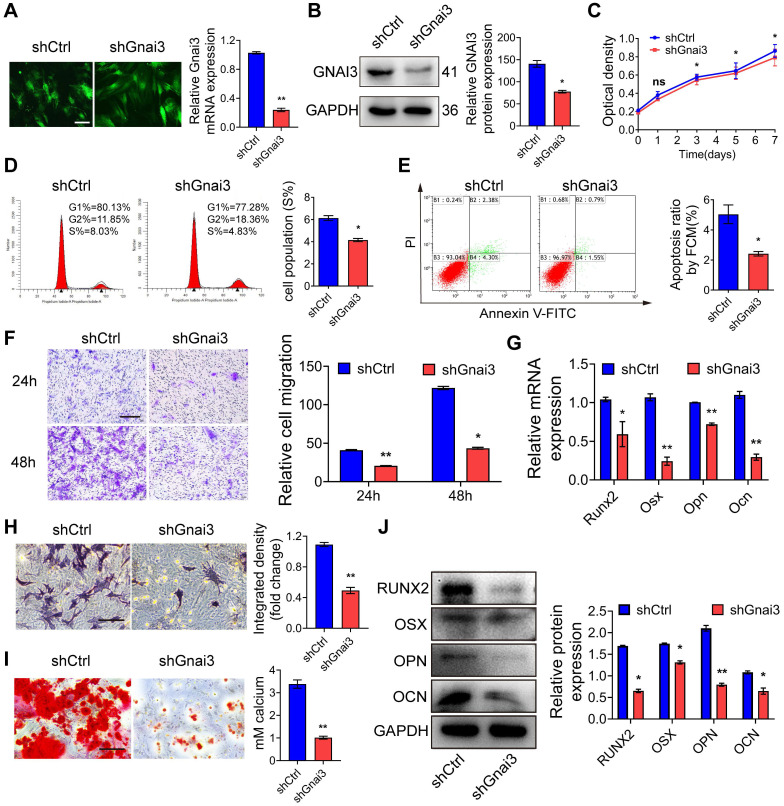
** Gnai3 stimulates osteogenic differentiation. (A)** Osteoblast precursor cells infected with lentivirus under fluorescence microscope, followed by qRT-PCR analysis performed to detect Gnai3 mRNA expression in shCtrl and shGnai3 osteoblast precursor cells. Bar = 100 μm. n = 4. **(B)** Efficiency of Gnai3 knockdown after infection with lentivirus analyzed by Western blotting. n = 4. **(C)** CCK-8 assay used to examine the proliferation of osteoblast precursor cells after infection with shGnai3 lentivirus. n = 5. **(D)** Cell cycle analysis performed by flow cytometry in shCtrl and shGnai3 groups. n = 4. **(E)** Apoptosis analysis performed by flow cytometry in shCtrl and shGnai3 groups. n = 4. **(F)** Transwell migration assay testing of osteoblast precursor cells at 24 and 48 hours in shCtrl and shGnai3 groups. Bar = 100 μm. n = 4. **(G)** Expression levels of osteogenic related genes (Runx2, Osx, Opn, Ocn) assessed by qRT-PCR. n = 4. **(H)** ALP staining observed after 5 days' mineralization. Bar = 100 μm. n = 4. **(I)** ARS staining observed after 14 days' mineralization. Bar = 100 μm. n = 4. **(J)** Expressions of osteogenic markers (RUNX2, OSX, OPN, OCN) assessed by Western blotting. n = 4. Data expressed as mean ± S.E.M. *p < 0.05, **p < 0.01, ns, not significant.

Osteoblast precursor cells were transduced with control shRNA (shCtrl) or Gnai3 shRNA (shGnai3) lentiviral particles, respectively, with MOI=50 to knockdown Gnai3. The transduction efficiency was estimated under a fluorescence microscope 72 h post-transduction **(Fig. 4A)**, and the knockdown efficiency was measured by both qRT-PCR and WB **(Fig. 4A, B)**. Then, CCK-8 and flow cytometry analyses were performed to determine the effect of Gnai3 expression on the osteoblast precursor cells proliferation **(Fig. 4C, D)**. We found that the osteoblast precursor cells proliferation in shGnai3 group was significantly decreased compared to that of shCtrl group, which was consistent with the phenotype of the Mimics group with overexpression of Mir24-2-5p, suggesting that Mir24-2-5p may play a negative regulatory role in Gnai3-mediated osteoblast precursor cells proliferation. Similarly, flow cytometry was performed to detect the effect of Gnai3 on the apoptosis of osteoblast precursor cells, which exhibited a significant reduction of apoptosis upon Gnai3 knockdown** (Fig. 4E)**. Next, a transwell migration assay was performed in shCtrl and shGnai3 treated osteoblast precursor cells to examine the effect of Gnai3 on the migration pattern of osteoblast precursor cells. The results showed that the migration of shGnai3 treated osteoblast precursor cells was significantly reduced **(Fig. 4F)**, which was consistent with the phenotype of the Mimics group. To explore the role of Gnai3 in osteoblast precursor cells differentiation, ALP staining, ARS staining and Von Kossa staining were performed after mineralization induction, which showed that the mineralization of the shGnai3 treated group was greatly reduced **(Fig. 4H, I; Fig. S1C)**. Analysis of osteoblast precursor cells differentiation-associated marker genes expression by qRT-PCR and WB revealed a significant reduction in differentiation after lentiviral shGnai3-mediated knockdown of Gnai3 **(Fig. 4G, J)**, which followed the trend in the phenotype of Mimics group, but was contradictory to that of the Inhibitor group, further confirming the negative regulation of Mir24-2-5p on Gnai3. Therefore, our experimental results suggested that Mir24-2-5p regulated the osteogenic differentiation of osteoblast precursor cells by targeting Gnai3 mRNA.

### Mir24-2-5p negatively regulates osteogenic differentiation by targeting Gnai3 *in vitro*

**Figure 5:**
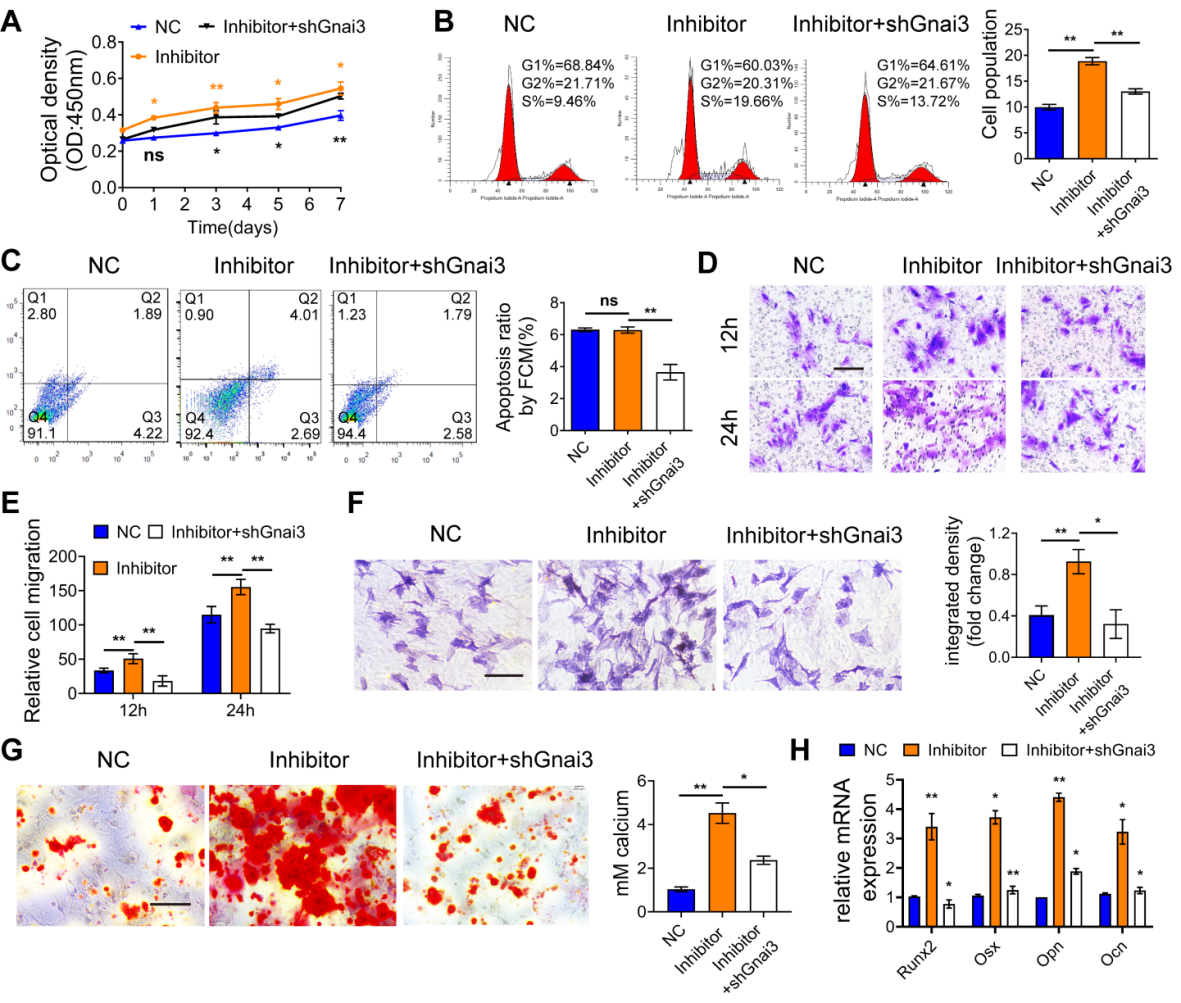
** Mir24-2-5p negatively regulates osteogenic differentiation by targeting Gnai3* in vitro*. (A)** CCK-8 assay used to examine the proliferation of osteoblast precursor cells co-transfected with Mir24-2-5p Inhibitor and shGnai3 lentivirus. n = 4. **(B)** Cell cycle analysis performed by flow cytometry in osteoblast precursor cells co-transfected with Mir24-2-5p Inhibitor and shGnai3 lentivirus. n=4. **(C)** Apoptosis analysis performed by flow cytometry in osteoblast precursor cells co-transfected with Mir24-2-5p Inhibitor and shGnai3 lentivirus. n=4. **(D)** Transwell migration assay testing of osteoblast precursor cells at 12 and 24 hours in osteoblast precursor cells co-transfected with Mir24-2-5p Inhibitor and shGnai3 lentivirus. Bar = 100 μm. **(E)** Quantitative analysis of Transwell migration assay. n = 4. **(F)** ALP staining observed after 5 days' mineralization. Bar = 100 μm. n = 4. **(G)** ARS staining observed after 14 days' mineralization. Bar = 100 μm. n = 4. **(H)** Expression levels of osteogenic related genes (Runx2, Osx, Opn, Ocn) assessed by qRT-PCR. n = 4. Data expressed as mean ± S.E.M. *p < 0.05, **p < 0.01, ns, not significant.

To verify the rescue effect of Gnai3 on cells with low expression level, osteoblast precursor cells were co-transfected with Mir24-2-5p inhibitor and shGnai3. Then, CCK-8 assay and flow cytometry were performed to detect the proliferation of osteoblast precursor cells, which indicated partial rescue of the abnormal cell proliferation **(Fig. 5A, B)**. Furthermore, osteoblast precursor cells apoptosis rate was decreased in these co-transfected cells compared with the Mir24-2-5p inhibitor alone treated group **(Fig. 5C)**, which was consistent with the previous findings **(Fig. 1D and Fig. 4D)**. Likewise, the abnormal cell migration in the Inhibitor alone treated group was partially rescued by knockdown of Gnai3 as measured by transwell migration assay** (Fig. 5D, E)**. Importantly, ALP and ARS staining and qRT-PCR assay for the expression of osteogenic differentiation marker genes confirmed that the abnormal osteogenic differentiation of the Inhibitor alone group was partially rescued by Gnai3 downregulation **(Fig. 5F-H)**. These results further confirmed our hypothesis that Mir24-2-5p might regulate the osteogenic differentiation of osteoblast precursor cells by negatively regulating Gnai3.

### *gnai3* mRNA partially rescues defective osteogenic differentiation phenotype in zebrafish embryos induced by *mir24-2-5p* overexpression

**Figure 6:**
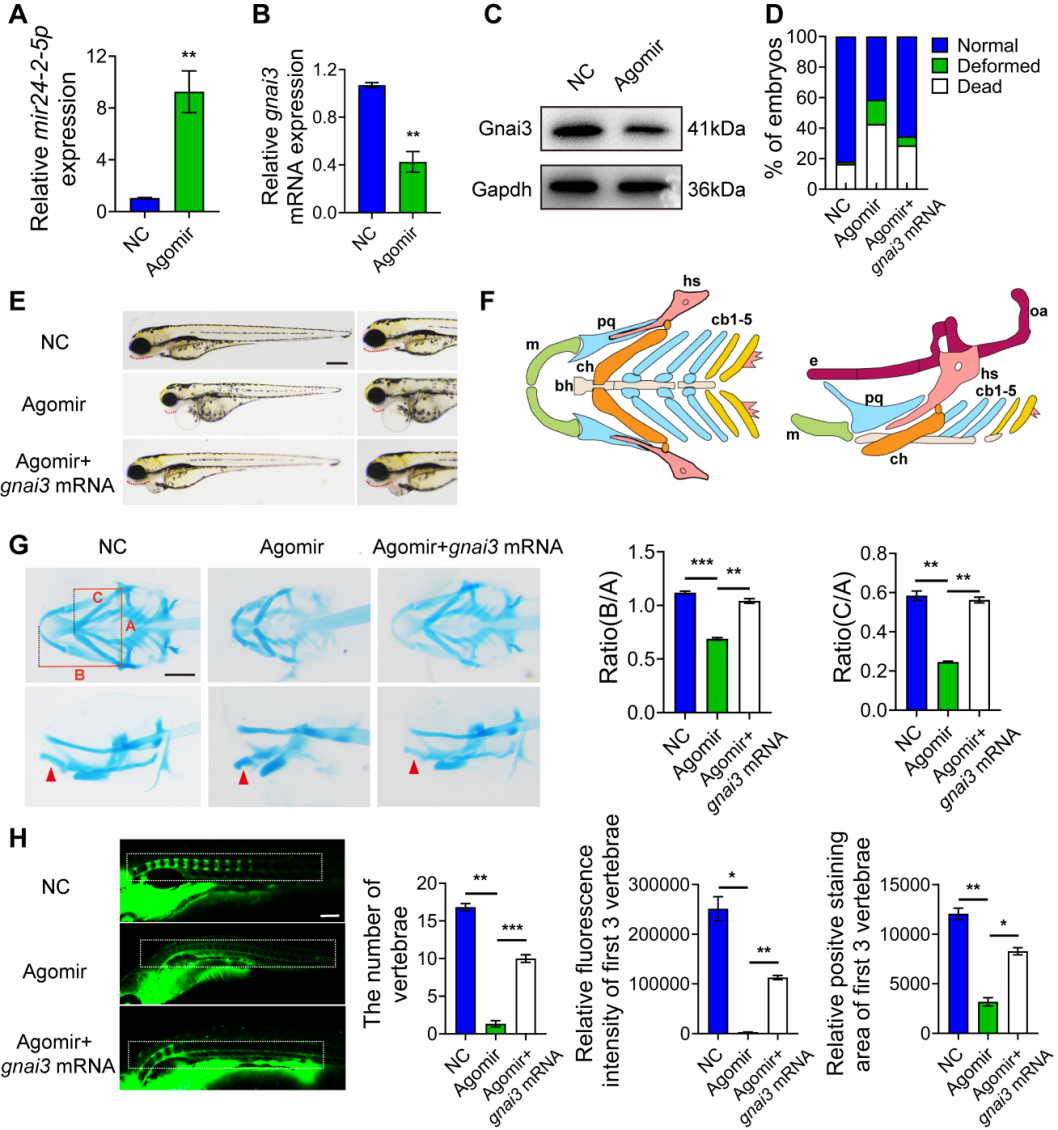
***mir24-2-5p* negatively regulates osteogenic differentiation through *gnai3* in zebrafish embryos.** *mir24-2-5p* expression level of embryos microinjected with *mir24-2-5p* Agomir confirmed by qRT-PCR. n = 6. **(B)** The *gnai3* mRNA expression level of embryos microinjected with *mir24-2-5p* Agomir detected by qRT-PCR. n = 6. **(C)** The Gnai3 protein expression level of embryos injected with *mir24-2-5p* Agomir detected by Western blotting. **(D)** Statistics of mortality and malformation rate in zebrafish embryos injected with *mir24-2-5p* Agomir and co-injected with *mir24-2-5p* Agomir + *gnai3* mRNA. n = 6. **(E)** Representative bright field images of zebrafish embryos injected with *mir24-2-5p* Agomir and co-injected with *mir24-2-5p* Agomir + *gnai3* mRNA at 96 hpf. hpf: hours post-fertilization. Red dotted line: mandible. Bar = 500 μm. **(F)** Schematic of the ventral (left) and lateral (right) view of the pharyngeal arch cartilage structure in zebrafish embryos at 120 hpf. m: Meckel's cartilage; bh: basihyal; ch: ceratohyal; pq: palatoquadrate; hs: hyosymplectic; cb: ceratobranchial; e: ethmoid plate; oa: occipital arch. **(G)** Ventral (upper row) and lateral view (lower row) of NC, Agomir, and Agomir + *gnai3* mRNA embryos by Alcian blue staining at 120 hpf. Red arrow, Meckel's cartilage. Bar = 100 μm. Quantification of the ratio of B/A or C/A followed. Line A served as a baseline for later measurements. Line B represented the distance from line A to the anterior of the Meckel's cartilage. Line C is the distance from line A to the anterior end of the ceratohyal. The ratio of line B divided by line A (B/A) and line C divided by line A (C/A) was quantified. n=6. (H) Visualization of calcified skeletal structures in NC, Agomir, and Agomir + *gnai3* mRNA embryos at 9 dpf by calcein staining. Bar = 200 μm. dpf: days post-fertilization. The quantification followed. n=6. Data expressed as mean ± S.E.M. *p < 0.05, **p < 0.01, ***p < 0.001, ns, not significant.

*mir24-2-5p* Agomir was microinjected into 1-2 cell phase of zebrafish embryos at the concentration of 2 ng/µl to facilitate *mir24-2-5p* overexpression. Then, RNA was extracted from zebrafish embryos at 24 h post-fertilization (hpf) for qRT-PCR analysis of *mir24-2-5p* level. The results showed that *mir24-2-5p* expression in zebrafish embryos was significantly increased by about 10-fold **(Fig. 6A)**, while *gnai3* mRNA and protein expression levels were subsequently reduced significantly **(Fig. 6B, C)**. As shown in **Fig. 6D** and **Fig. S1D**, malformation rate of zebrafish embryos was increased as counted 24 h after* mir24-2-5p* Agomir microinjection, which was rescued by co-injection with *gnai3* mRNA. Consistently, Agomir-treated embryos exhibited growth retardation and significant deformity with shorter body size and retracted mandibular compared with that of the NC group at 96 hpf, which were partially rescued by *gnai3* mRNA **(Fig. 6E)**. Hence, Alcian blue staining which was performed to detect craniofacial cartilage development of zebrafish embryos at 120 hpf, which revealed that both the angles of the hyoid cartilage and Meckel cartilage were increased, resulting in insufficient mandibular development and mandibular retrusion. This defective phenotype was also rescued by co-injecting with *gnai3* mRNA **(Fig. 6F, G)**. For further verification of vertebral calcification, zebrafish embryos were stained with calcein at 9 dpf, which showed that the number of calcified vertebrae, the fluorescence intensity, and the positive staining area of the first 3 vertebrae in Agomir group were significantly reduced compared with those in the NC group, which were partially rescued by microinjection of *gnai3* mRNA **(Fig. 6H)**. To sum up, *mir24-2-5p* overexpression resulted in defects of craniofacial development and osteogenic differentiation in zebrafish embryos, which could be repaired by *gnai3* overexpression.

### *mir24-2-5p* inhibits osteogenic differentiation by targeting* gnai3* in zebrafish embryos

**Figure 7:**
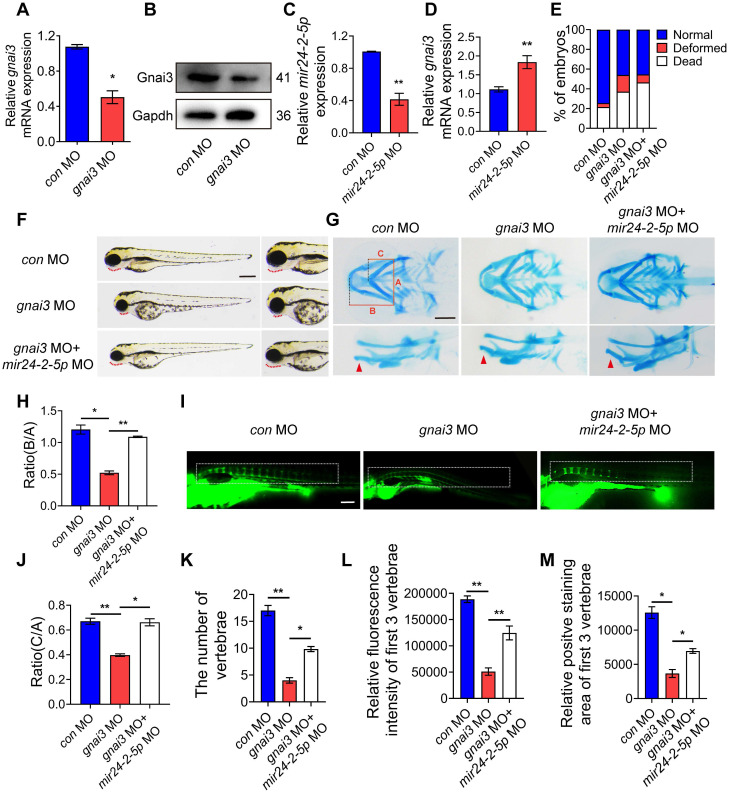
***mir24-2-5p* inhibits osteogenic differentiation by targeting *gnai3* in zebrafish embryos. (A)** *gnai3* mRNA knockdown efficiency in the *gnai3* MO embryos confirmed by qRT-PCR. MO: morpholino. n=6. **(B)** The expression level of Gnai3 protein in the *gnai3* MO embryos detected by Western blotting. **(C)** The expression level of *mir24-2-5p* in *mir24-2-5p* MO embryos detected by qRT-PCR. n=6. **(D)** The expression level of *gnai3* mRNA in *mir24-2-5p* MO embryos detected by qRT-PCR. n=6. **(E)** Statistics of mortality and malformation rate in zebrafish embryos injected with *gnai3* MO and co-injected with *gnai3* MO + *mir24-2-5p* MO. n = 6. **(F)** Bright-field images of zebrafish embryos injected with *gnai3* MO and co-injected with *gnai3* MO + *mir24-2-5p* MO. at 96 hpf. hpf, hours post-fertilization. Red dotted line: mandible. Bar = 500 μm. **(G)** Ventral (upper row) and lateral view (lower row) of *con* MO, *gnai3* MO and *gnai3* MO + *mir24-2-5p* MO embryos by Alcian blue staining at 120 hpf. Red arrow, Meckel's cartilage. Bar = 100 μm. **(H-I)** Quantification of the ratio of B/A or C/A of Alcian blue staining. n=6. **(J)** Visualization of calcified skeletal structures in *con* MO, *gnai3* MO and *gnai3* MO + *mir24-2-5p* MO embryos at 9 dpf by calcein staining. dpf, days post-fertilization. Bar = 200 μm. **(K-M)** The quantification of calcein staining. n=6. Data expressed as mean ± S.E.M. *p < 0.05, **p < 0.01.

*gnai3* MO was microinjected into zebrafish embryos to inhibit *gnai3* expression, resulting in ~50 % knockdown as measured by qRT-PCR as well as WB **(Fig. 7A, B)**. At the same time, *mir24-2-5p* MO was microinjected into zebrafish embryos to inhibit *mir24-2-5p* expression, resulting in ~60 % knockdown as measured by qRT-PCR** (Fig. 7C)**. As expected, *gnai3* mRNA expression was increased in *mir24-2-5p* MO, which is confirmed by qRT-PCR **(Fig. 7D)**. Interestingly, the malformation rate of *gnai3* MO were significantly increased at 24 hpf, which was rescued by co-injection with *mir24-2-5p* MO** (Fig. 7E, Fig. S1E)**. Compared with the control group, *gnai3* MO treated group exhibited growth retardation with significant deformity at 96 hpf, such as shorter body size and retracted mandibular, which was quite similar to Agomir group with *mir24-2-5p* overexpression, and the phenotype was also rescued by *mir24-2-5p* MO **(Fig. 7F)**. Similarly, alcian blue staining at 120 hpf showed that both the hyoid cartilage and Meckel cartilage deficiency were occurred in *gnai3* MO embryos, which were partially saved by *mir24-2-5p* MO **(Fig. 7G-I)**. Moreover, calcein staining exhibited that the number of calcified vertebrae, the fluorescence intensity and positive staining area of first 3 vertebrae in *gnai3* MO group were significantly reduced compared to *control* MO, which was also compensated by *mir24-2-5p* MO **(Fig. 7J-M)**. Taken together, these results suggested that *gnai3* plays an important role in the regulation of cartilage development and vertebral calcification in zebrafish embryos, and *mir24-2-5p* functionally and directly prevented *gnai3* from promoting osteogenic differentiation.

### Mir24-2-5p negatively regulates Gnai3 in osteogenic differentiation of osteoblast precursor cells via MAPK signaling

**Figure 8:**
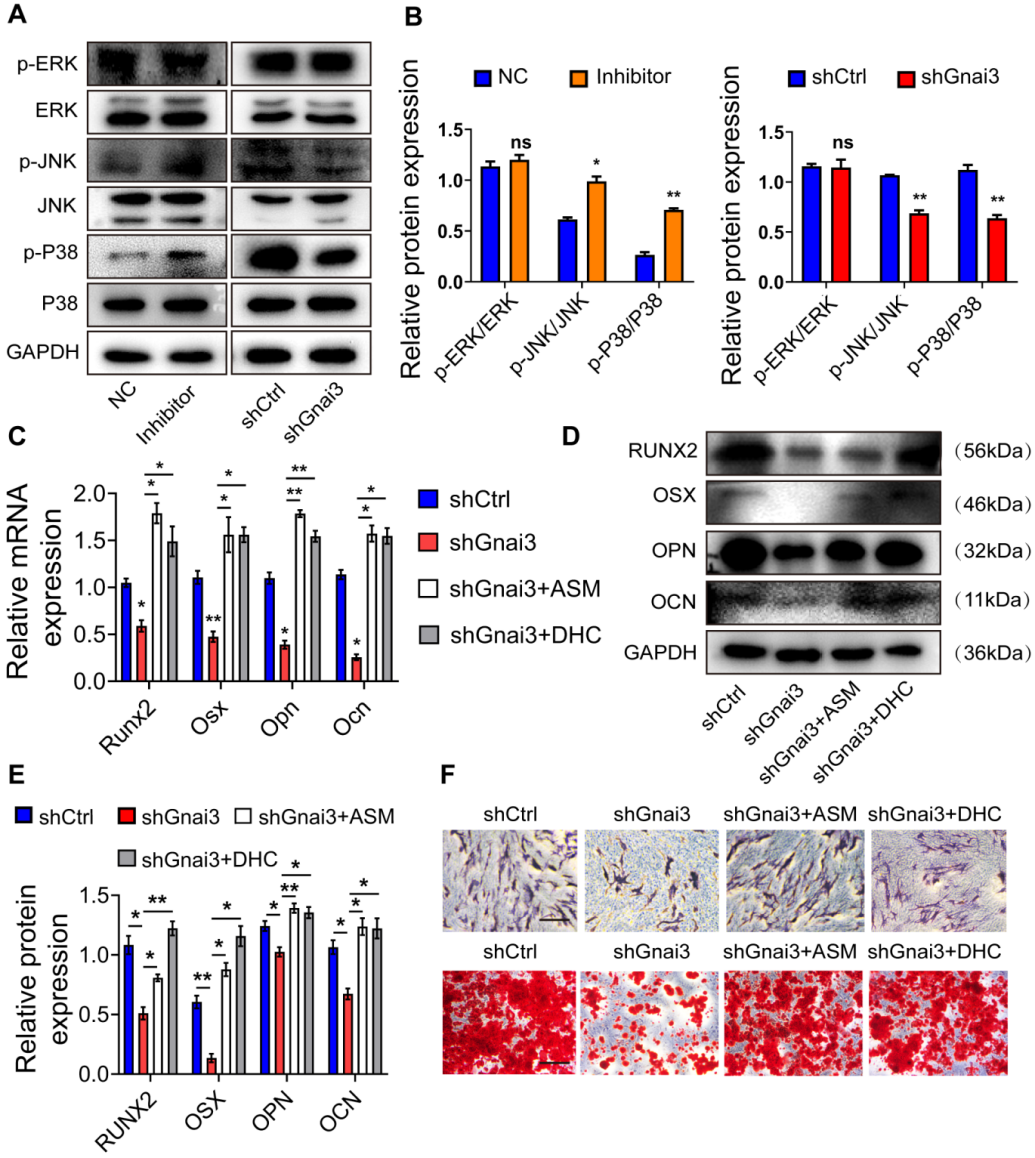
** Mir24-2-5p negatively regulates Gnai3 in osteogenic differentiation of osteoblast precursor cells via MAPK signal pathway. (A)** The protein expression of p-ERK, p-JNK and p-p38 levels detected by Western blotting. **(B)** Quantitative analysis of WB. of p-ERK, p-JNK and p-p38. n=3. **(C)** qRT-PCR assay carried out for osteogenic differentiation marker genes in shCtrl, shGnai3, shGnai3+ASM and shGnai3+DHC groups. n=4. **(D)** WB performed to detect expression of osteogenic differentiation marker proteins in shCtrl, shGnai3, shGnai3+ASM and shGnai3+DHC groups. **(E)** Quantitative analysis of WB of RUNX2, OSX, OPN and OCN. n=3. **(F)** ALP and ARS staining in shCtrl, shGnai3, shGnai3+ASM and shGnai3+DHC groups. Bar = 100 μm. Data expressed as mean ± S.E.M. *p < 0.05, **p < 0.01, ns, not significant.

Mitogen-activated protein kinase (MAPK) signaling is a critical regulator of the osteoblast precursor cells differentiation process. Therefore, WB was performed to detect the expressions of p-ERK/ERK, p-JNK/JNK, p-p38/p38 both in the Inhibitor and shGnai3 groups. Interestingly, there was no significant change in levels of p-ERK between NC and Mir24-2-5p Inhibitor group, but the expressions of p-JNK and p-p38 were increased significantly in the Mir24-2-5p Inhibitor group. Moreover, there were no significant changes in p-ERK levels between shCtrl and shGnai3 groups, but the expressions of both p-JNK and p-p38 decreased significantly in the shGnai3 group **(Fig. 8A, B)**. These results together suggest that MAPK signaling may be involved in the regulation of Mir24-2-5p and Gnai3 functions during the osteoblast precursor cells differentiation. For further verification, p-JNK activator anisomycin (ASM) and p-p38 activator dehydrocorydaline chloride (DHC) were added to shGnai3 osteoblast precursor cells, respectively, followed by qRT-PCR and WB assays for determining the mRNA **(Fig. 8C)** and protein levels **(Fig. 8D, E)** of osteogenic differentiation markers. The osteogenic differentiation was improved in osteoblast precursor cells treated with ASM/DHC compared with the mock-treated shGnai3 group. ALP and ARS staining showed similar results **(Fig. 8F)**. Together, these results confirmed that Mir24-2-5p might regulate osteoblast precursor cells differentiation through regulating the Gnai3-p-JNK/p-p38 signaling axis.

## Discussion

In this study, we initially found that during the mineralization process of osteoblast precursor cells, the expression of Mir24-2-5p was significantly decreased over time. Hsa-miR-24-5p (previous ID: hsa-miR-24-2*) and mmu-Mir24-2-5p (previous ID: mmu-miR-24-2*) share highly conserved sequences, and accumulated reports highlighted the expression profiles of Mir24-2-5p in multiple cellular processes, including cancer, stemness, and stress responses 20-22. However, only a handful of studies have explored the regulatory roles of Mir24-2-5p in the osteogenesis signaling network, in addition, the presented results are not sufficient to pinpoint its exact mechanistic functions. Interestingly, 10 plasma microRNAs, including Mir24-2-5p, have been found to have a direct relation with osteoporotic fractures 13. Besides, our previous work showed 49 downregulated serum miRNAs, including Mir24-2-5p, in young individuals with the overgrowing mandible (mandibular protrusion) with at least a two-fold difference by miRNA microarray assay 15. To explore the possible roles of Mir24-2-5p in the osteogenic process, the cellular effects of its knockdown and overexpression were investigated. The cell proliferation, migration, osteogenic differentiation, and mineralization examinations showed a reverse correlation with the expression of Mir24-2-5p, suggesting that Mir24-2-5p negatively regulated osteoblast precursor cells differentiation.

Some ACS cases have been characterized by apparent abnormal development of the mandible: instead of developing normally, the lower jaw shaped more like, the smaller upper jaw (maxilla). This abnormal shape led to an unusually small chin (micrognathia) and problems with jaw function 23. Genomic alterations in PLCB4, GNAI3, and EDN1 genes have been identified to be responsible for >90 % of ACS onsets. In this context, it is interesting to note that all these genes have been implicated in endothelin signaling, which plays a critical role as a mediator promoting bone matrix synthesis and mineralization 24-26. It has been reported that conditional knockdown of GATA4 gene causes shorter tooth root and inhibits odonto/osteogenesis. Notably, GNAI3 has been identified as one of these disease-related proteins after GATA4 deletion by isobaric tags for relative and absolute quantification (iTRAQ) method 27. Accordingly, we inferred that Gnai3 was closely related to osteogenesis processes, and our previous research on the role of Gnai3 in odontogenesis and osteogenesis in stem cells from the apical papilla preliminary supported this hypothesis 8. In this study, both *in vitro* and *in vivo* knockdown of Gnai3 were conducted, and the obtained results were in agreement with our hypothesis. Silencing of Gnai3 inhibited osteoblast precursor cells proliferation, migration, osteogenic differentiation, and mineralization. Embryo microinjection of *gnai3* MO resulted in shorter body length, smaller and retruded mandible, poorer cartilage development, and decreased vertebral calcification. Reverse influence profiles of Mir24-2-5p and Gnai3 in osteogenesis were revealed, however, their possible cross-talk was remained elusive.

Furthermore, silencing of Gnai3 showed a similar phenotype with overexpression of Mir24-2-5p. Moreover, in Mir24-2-5p downregulated osteoblast precursor cells, the expression of Gnai3 was significantly increased, suggesting that Mir24-2-5p might negatively target Gnai3. Dual-luciferase reporter gene assay further confirmed the combinatorial effects of Mir24-2-5p and Gnai3. Secondly, rescue experiments for Gnai3 expression under both *in vitro* and* in vivo* conditions were conducted. Promoting effects of osteoblast precursor cells in proliferation, migration, osteogenic differentiation, and mineralization by low expression of Mir24-2-5p were partially rescued upon silencing of Gnai3. Phenotype of osteogenic differentiation defects in zebrafish embryos caused by *mir24-2-5p* Agomir and *gnai3* MO were partially rescued by *gnai3* mRNA and *mir24-2-5p* MO respectively. However, the molecular mechanisms underlying this process remain generally indefinite. A multitude of signaling pathways regulates the intricate osteoblast precursor cells differentiation network, such as Wnt, Notch, and MAPK signaling pathways 28, 29. Many osteogenesis-related extracellular signaling pathways such as BMP/Smad, TGF, and TNF are transduced through cascades of multilevel protein kinases in MAPK pathway to trigger intracellular biological responses 30-33. Low expression of Mir24-2-5p promoted the phosphorylation of JNK and p38, but not ERK in MAPK signaling; On the contrary, low expression of Gnai3 inhibited the phosphorylation of JNK and p38, but not ERK in MAPK. These reverse expression patterns suggest that MAPK signaling pathway may be involved in the regulation of inhibition of Mir24-2-5p targeted upregulation Gnai3 expression during osteoblast precursor cells differentiation. Furthermore, osteogenic differentiation-associated phosphorylation of JNK and p38 activity was first significantly inhibited by low expression of Gnai3 and then greatly boosted by p-JNK and p-p38 pathway activators, respectively. JNK MAPK has been identified as a late-stage positive regulator of osteoblast precursor cells differentiation, and p38 MAPK is similar to JNK MAPK in that it responds to a multitude of stimuli and is recognized as the biomarker for the early stage of osteoblast precursor cells differentiation 34, 35. In our study, we demonstrated that Mir24-2-5p negatively regulated osteoblast precursor cells differentiation by partly inactivating the Gnai3-p-JNK/p-p38 signaling axis **(Graphical Abstract)**.

In summary, we reported a new mechanism of interaction between Mir24-2-5p and Gnai3, at both transcriptional and post-transcriptional levels, in relation to osteogenesis. Inhibition of Mir24-2-5p targeted upregulation of Gnai3 resulted in promotion of osteoblast precursor cells osteogenic differentiation and mandibular development through activating MAPK (p-JNK/p-p38) signaling cascades. Our findings thus indicated that Mir24-2-5p might provide an attractive diagnostic method and therapeutic intervention for treating congenital bone defects and craniofacial developmental deformities.

## Supplementary Material

Supplementary figure and table.

## Figures and Tables

**Table A:** Thermal cycling profile

Stage		Reps	Temp.	Time
Stage 1	Pre-denaturation	1	95 °C	30 s
Stage 2	Cycle reaction	40	95 °C	10 s
			60 °C	30 s
Stage 3	Dissolution curve	1	95 °C	15 s
			60 °C	60 s
			95 °C	15 s
			60 °C	15 s

## References

[1] Zha JP, Wang XQ, Di J (2020). MiR-920 promotes osteogenic differentiation of human bone mesenchymal stem cells by targeting HOXA7. J Orthop Surg Res.

[2] Passos-Bueno MR, Ornelas CC, Fanganiello RD (2009). Syndromes of the first and second pharyngeal arches: A review. Am J Med Genet A.

[3] Papagrigorakis MJ, Karamolegou M, Vilos G (2012). Auriculo-condylar syndrome. Angle Orthod.

[4] Tu Y, Qu T, Chen F (2019). Mutant hFGF23(A12D) stimulates osteoblast precursor cells differentiation through FGFR3. J Cell Mol Med.

[5] Zhu H, Ge K, Lu J (2020). Downregulation of GNAI3 Promotes the Pathogenesis of Methionine/Choline-Deficient Diet-Induced Nonalcoholic Fatty Liver Disease. Gut Liver.

[6] Williams SE, Ratliff LA, Postiglione MP (2014). Par3-mInsc and Gαi3 cooperate to promote oriented epidermal cell divisions through LGN. Nat Cell Biol.

[7] Ren YM, Zhao X, Yang T (2018). Exploring the Key Genes and Pathways of Osteoarthritis in Knee Cartilage in a Rat Model Using Gene Expression Profiling. Yonsei Med J.

[8] Zhang Y, Yuan L, Meng L (2019). Guanine and nucleotide binding protein 3 promotes odonto/osteogenic differentiation of apical papilla stem cells via JNK and ERK signaling pathways. Int J Mol Med.

[9] Rieder MJ, Green GE, Park SS (2012). A human homeotic transformation resulting from mutations in PLCB4 and GNAI3 causes auriculocondylar syndrome. Am J Hum Genet.

[10] Ruest LB, Xiang X, Lim KC (2004). Endothelin-A receptor-dependent and -independent signaling pathways in establishing mandibular identity. Development.

[11] Miller CT, Yelon D, Stainier DY (2003). Two endothelin 1 effectors, hand2 and bapx1, pattern ventral pharyngeal cartilage and the jaw joint. Development.

[12] Peng S, Gao D, Gao C (2016). MicroRNAs regulate signaling pathways in osteogenic differentiation of mesenchymal stem cells (Review). Mol Med Rep.

[13] Verdelli C, Sansoni V, Perego S (2020). Circulating fractures-related microRNAs distinguish primary hyperparathyroidism-related from estrogen withdrawal-related osteoporosis in postmenopausal osteoporotic women: A pilot study. Bone.

[14] Seeliger C, Karpinski K, Haug AT (2014). Five freely circulating miRNAs and bone tissue miRNAs are associated with osteoporotic fractures. J Bone Miner Res.

[15] Gu Y, Zhang Y, Zhao C (2014). Serum microRNAs as potential biomarkers of mandibular prognathism. Oral Dis.

[16] Wang D, Weng Y, Guo S (2019). microRNA-1 Regulates NCC Migration and Differentiation by Targeting sec63. Int J Biol Sci.

[17] Liu C, Wang X, Zhang H (2015). Immortalized Mouse Floxed Fam20c Dental Papillar Mesenchymal and Osteoblast Cell Lines Retain Their Primary Characteristics. J Cell Physiol.

[18] Lau MC, Kwong EM, Lai KP (2016). Pathogenesis of POLR1C-dependent Type 3 Treacher Collins Syndrome revealed by a zebrafish model. Biochim Biophys Acta.

[19] Goudevenou K, Martin P, Yeh YJ (2011). Def6 is required for convergent extension movements during zebrafish gastrulation downstream of Wnt5b signaling. PLoS One.

[20] Yang X, Wang L, Li R (2018). The long non-coding RNA PCSEAT exhibits an oncogenic property in prostate cancer and functions as a competing endogenous RNA that associates with EZH2. Biochem Biophys Res Commun.

[21] Lee SH, Chen TY, Dhar SS (2016). A feedback loop comprising PRMT7 and miR-24-2 interplays with Oct4, Nanog, Klf4 and c-Myc to regulate stemness. Nucleic Acids Res.

[22] Chen RJ, Kelly G, Sengupta A (2015). MicroRNAs as biomarkers of resilience or vulnerability to stress. Neuroscience.

[23] Gordon CT, Cunniff CM, Green GE (2014). Clinical evidence for a mandibular to maxillary transformation in Auriculocondylar syndrome. Am J Med Genet A.

[24] Clouthier DE, Passos-Bueno MR, Tavares AL (2013). Understanding the basis of auriculocondylar syndrome: Insights from human, mouse and zebrafish genetic studies. Am J Med Genet C Semin Med Genet.

[25] Nabil A, El Shafei S, El Shakankiri NM (2020). A familial PLCB4 mutation causing auriculocondylar syndrome 2 with variable severity. Eur J Med Genet.

[26] Kristianto J, Johnson MG, Afzal R (2017). Endothelin Signaling in Bone. Endocrinol Metab Clin North Am.

[27] Guo S, Zhang Y, Zhou T (2017). Role of GATA binding protein 4 (GATA4) in the regulation of tooth development via GNAI3. Sci Rep.

[28] Maeda K, Kobayashi Y, Koide M (2019). The Regulation of Bone Metabolism and Disorders by Wnt Signaling. Int J Mol Sci.

[29] Luo Z, Shang X, Zhang H (2019). Notch Signaling in Osteogenesis, Osteoclastogenesis, and Angiogenesis. Am J Pathol.

[30] Zhang Y, Gu X, Li D (2019). METTL3 Regulates Osteoblast Differentiation and Inflammatory Response via Smad Signaling and MAPK Signaling. Int J Mol Sci.

[31] Li H, Zhang S, Nie B (2019). KR-12-a5 Reverses Adverse Effects of Lipopolysaccharides on HBMSC Osteogenic Differentiation by Influencing BMP/Smad and p38 MAPK Signaling Pathways. Front Pharmacol.

[32] Sun X, Xie Z, Ma Y (2018). TGF-beta inhibits osteogenesis by upregulating the expression of ubiquitin ligase SMURF1 via MAPK-ERK signaling. J Cell Physiol.

[33] Zhang Y, Yang C, Ge S (2020). EphB4/ TNFR2/ERK/MAPK signaling pathway comprises a signaling axis to mediate the positive effect of TNF-alpha on osteogenic differentiation. BMC Mol Cell Biol.

[34] Xu R, Zhang C, Shin DY (2017). c-Jun N-Terminal Kinases (JNKs) Are Critical Mediators of Osteoblast Activity *in vivo*. J Bone Miner Res.

[35] Kwak SC, Jeong DH, Cheon YH (2020). Securinine suppresses osteoclastogenesis and ameliorates inflammatory bone loss. Phytother Res.

