# Supplementary material for: Mir24-2-5p suppresses the osteogenic differentiation with Gnai3 inhibition presenting a direct target via inactivating JNK-p38 MAPK signaling axis
Source: Int J Biol Sci. 2021 Oct 17;17(15):4238–53. doi: 10.7150/ijbs.60536 (PMC8579458; doi:10.7150/ijbs.60536)
Supplement: Supplementary file 1 — Supplementary figure and table. [file ijbsv17p4238s1.pdf]

Supplementary Information

Mir24-2-5p suppresses the osteogenic differentiation with Gnai3 inhibition presenting a direct target via inactivating JNK-p38 MAPK signaling axis

Li Meng, Lichan Yuan, Jieli Ni, Mengru Fang, Shuyu Guo, Jinwei Qin, Qi Cai, Mengnan Zhang, Fang Hu, Junqing Ma, Yang Zhang

Figure S1

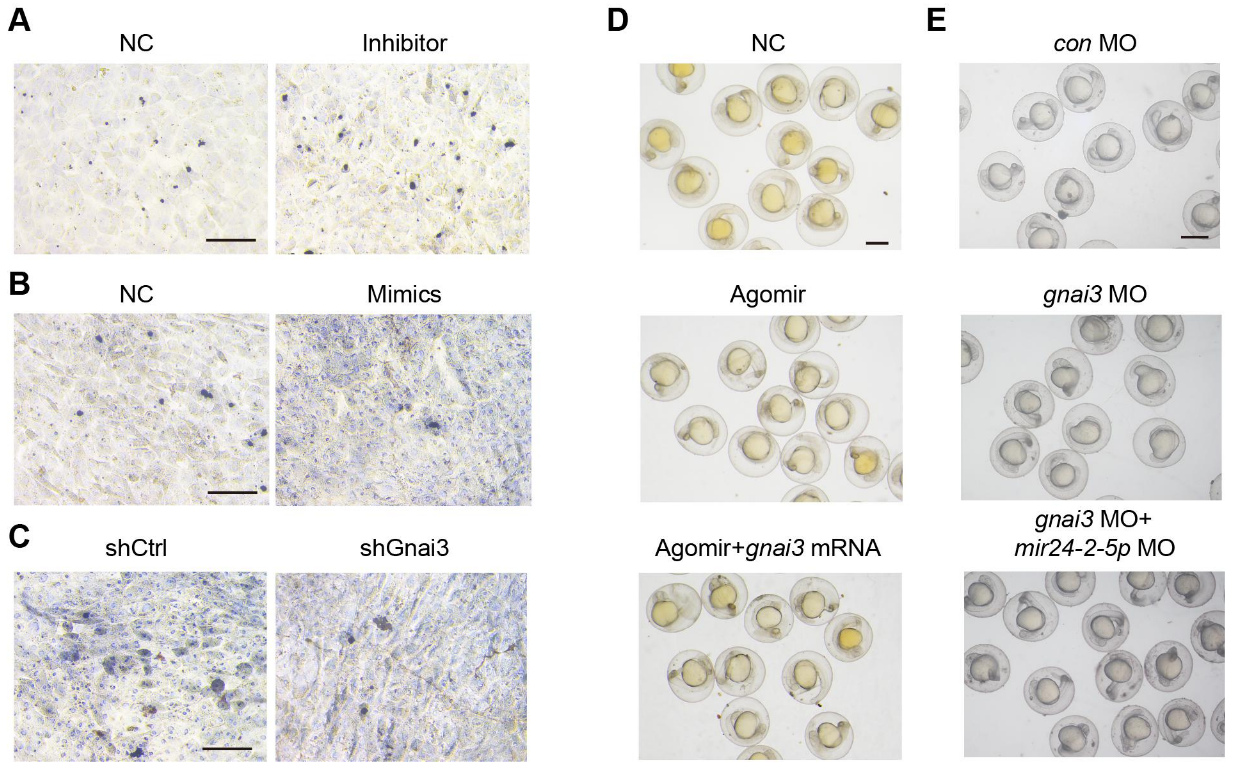

Figure S1: Von Kossa staining and images of zebrafish embryos at 24 hpf. (**A**) Von Kossa staining of cells transfected Mir24-2-5p Inhibitor. Bar=100μm. (**B**)Von Kossa staining of cells transfected Mir24-2-5p Mimics. Bar=100μm. (**C**)Von Kossa staining of shCtrl and shGnai3 cells. Bar=100μm. (**D**) Bright-field images of zebrafish embryos injected with *mir24-2-5p* NC, *mir24-2-5p* Agomir and *mir24-2-5p* Agomir+ *gnai3* mRNA at 24 hpf. Bar = 200 μm. hpf, hours post-fertilization. (**E**) Bright-field images of zebrafish embryos injected with *con* MO, *gnai3* MO and *gnai3* MO+ *mir24-2-5p* MO at 24 hpf. Bar = 200 μm. hpf, hours post-fertilization.

Table S1

The primers used in this research.

Gene	Forward	Reverse
mus musculus-Gapdh	ACCACAGTCCATGCCATCAC	TCCACCACCCTGTTGCTGTA
mus musculus-U6	TCGGCAGCACATATACTAAAATTG	CTTCACGAATTTGCGTGTCAT
mus musculus-Mir24-2-5p	CCGCATTGGCTCAGTTCAG	TATGGTTGTTGACGACTGGTTGAC
mus musculus-Runx2	AAGTGTTCTGTGGTCTCTGAGTTGA	GCTGTATGGTGAGGCTGGTAGG
mus musculus-Osx	GCTGGGTAGAGGAAGGTC	CAAAGAGAGAAGAACTGAGGAG
mus musculus-Opn	ACCATGCAGAGAGCGAGGATT	GGGACATCGACTGTAGGGACG
mus musculus-Ocn	ATCTTTCTGCTCACTCTGCT	CTACCTTATTGCCCTCCTG
mus musculus-Gnai3	GATATTCCCTGGACCTGCTAA	AGATGATGCCCGACAGTTATT
danio rerio-*gapdh*	ACCACAGTCCATGCCATCAC	TCCACCACCCTGTTGCTGTA
danio rerio-*U6*	CAGCACATATACTAAAATTGGAACG	ACGAATTTGCGTGTCATCC
danio rerio-*mir24-2-5p*	ACCGCATTTGTGCCTACTGAG	TATGGTTGTTGACGACTGGTTGAC
danio rerio-*gnai3*	AGGTCAAACTCCTGCTGCTC	TGTTGCTGTAAACCACCACTT
